# Rare case of schwannomatosis presenting with cauda equina syndrome: a case report

**DOI:** 10.1097/MS9.0000000000003268

**Published:** 2025-04-10

**Authors:** Allahdad Khan, Nehan Zahoor, Abdul Ahad Riaz, Humaira Siddique, Anam Malik, Raheel Ahmed, Fnu Poombal, Mohamed Antar

**Affiliations:** a Department of Medicine, Nishtar Medical University, Multan, Pakistan; b Department of Neurosurgery, Nishtar Medical University, Multan, Pakistan; c Department of Radiology, Nishtar Medical University, Multan, Pakistan; d Department of Pathology, Nishtar Medical University, Multan, Pakistan; e National Heart and Lung Institute, Imperial College London, UK; f Department of Pathology, UMass Chan Medical School - Baystate Regional Campus, Springfield, Massachusetts, USA; g Faculty of Medicine, Tishreen University Faculty of Medicine, Latakia, Syrian Arab Republic

**Keywords:** case report, cauda equina syndrome, nerve sheath tumors, schwannomatosis, spinal schwannoma

## Abstract

**Background:**

Schwannomatosis is a rare disorder characterized by multiple schwannomas without vestibular schwannomas or other features of neurofibromatosis type 2 (NF2). It commonly presents with neuropathic pain, neurological deficits, and soft tissue tumors but rarely leads to cauda equina syndrome, a serious condition requiring urgent intervention.

**Materials and methods:**

We report a 28-year-old Pakistani female with progressive back pain, lower limb weakness, sensory deficits, bladder and bowel incontinence, and multiple tender swellings, consistent with cauda equina syndrome. Neurological examination revealed right-sided lower limb weakness. MRI of the brain showed no vestibular schwannomas, while spinal MRI identified a heterogeneously enhancing schwannoma from D11 to L5 with cystic extension into the neural foramina, proximal central canal dilation, and spinal cord compression. Multiple additional schwannomas were detected. Right thigh mass biopsy confirmed schwannoma, showing Antoni A and B regions, Verocay bodies, and S-100 positivity. Genetic testing was not performed due to financial constraints.

**Results:**

The patient underwent partial spinal schwannoma resection, leading to spinal decompression and resolution of cauda equina syndrome symptoms. Symptomatic cutaneous schwannomas were excised. Neuropathic pain was managed with pregabalin and NSAIDs. At 6-month follow-up, she showed improved lower limb strength, resolution of incontinence, and no significant tumor regrowth.

**Conclusion:**

This case highlights schwannomatosis presenting with cauda equina syndrome, emphasizing the importance of early recognition, spinal decompression, and differentiation from NF2 for optimal management.

## Introduction

Schwannomatosis represents a distinctive neurofibromatosis condition which causes multiple schwannomas while sparing vestibular schwannomas together with the other NF2-related features^[1]^. Schwannomatosis is a rare disorder with an estimated incidence ranging from 1 in 40 000 to 1 in 1.7 million. Patients typically develop this condition within a range of 30–60 years of age with no identified correlation to race or gender^[2,3]^. Here we report a rare case of schwannomatosis leading to cauda equina syndrome in a 28-year-old female. This case report has been reported according to the SCARE guidelines^[4]^.

**HIGHLIGHTS**

- Schwannomatosis is a distinct form of neurofibromatosis characterized by multiple schwannomas without vestibular schwannomas or other features of NF2.
- Cauda equina syndrome is a rare but serious complication of schwannomatosis leading to lower limb weakness, sensory deficits, bladder, and bowel dysfunction.
- MRI findings revealed a heterogeneously enhancing schwannoma extending from D11 to L5, with cystic extension into neural foramina, proximal central canal dilation, and multiple additional schwannomas.
- Surgical intervention with partial spinal schwannoma resection led to resolution of cauda equina symptoms and improved lower limb strength.

## Case presentation

A 28 years old female of Pakistani origin presented to outpatient department with complaints of worsening back pain radiating to bilateral lower limbs for 1 year associated with numbness and weakness in legs, bladder and bowel incontinence, discomfort in her right arm, and multiple tender swellings over her body. The patient had no family history of such a condition and was currently on no medication. On initial presentation, the patient was alert and oriented with a Glasgow Coma Scale of 15/15. Pupils were bilaterally reactive to light, and plantar reflexes were bilaterally downgoing, indicating the absence of upper motor neuron involvement.

Neurological examination revealed decreased sensation and motor weakness in the lower limbs, more pronounced on the right side, with muscle strength graded 3/5 on the Medical Research Council (MRC) scale. Hyporeflexia was noted in the patellar and Achilles reflexes, with absent bulbocavernosus reflex and loss of voluntary anal sphincter contraction, consistent with cauda equina syndrome.

In addition, multiple tender, firm, and mobile masses were palpated on the left lower abdominal wall, right arm, right thigh, and bilaterally on the anterior and posterior chest wall. However, cutaneous features of neurofibromatosis, such as café-au-lait spots, were absent, further supporting a diagnosis of schwannomatosis.

**Figure 1.**
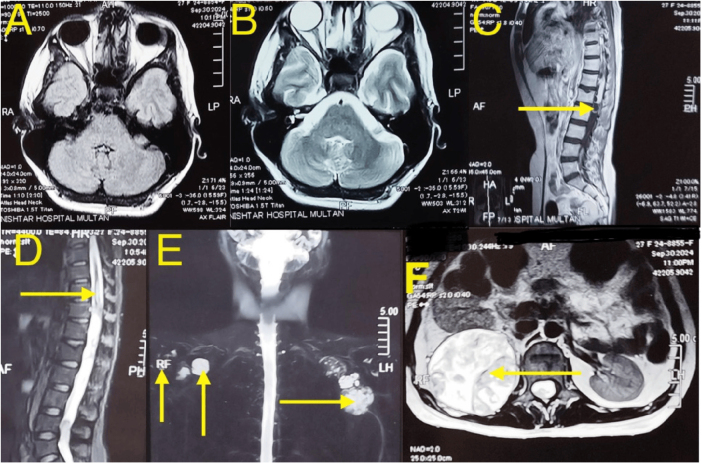
MRI brain plain, axial sections FLAIR sequence (A) and T2W sequence (B), show no abnormal MR signal intensity lesion (vestibular schwannoma) at bilateral cerebello-pontine angles. Post-contrast MRI thoracolumbar spine, Sagittal section T1W sequence with contrast (C), shows heterogeneously enhancing solid cystic intramedullary extradural lesion seen opposite D11 to L5 levels. MRI thoracolumbar spine, Sagittal section T1W with fat suppression (D), shows hyperintense abnormal MR signals returning from cord opposite D10–D11 vertebral levels. Post-contrast MRI spine, coronal section, myelogram (E), shows solid enhancing lesions along bilateral anterior chest and medical aspect of right upper arm. Post-contrast MRI spine, T1W sequence with contrast, axial section (F), showing solid enhancing lesion in right lumbar region.

MRI (Fig. 1) revealed a large, heterogeneously enhancing schwannoma extending from D11 to L5, with cystic extension into the neural foramina and cord compression. Additional schwannomas were noted in the thoracic, lumbar, and peripheral regions.

**Figure 2.**
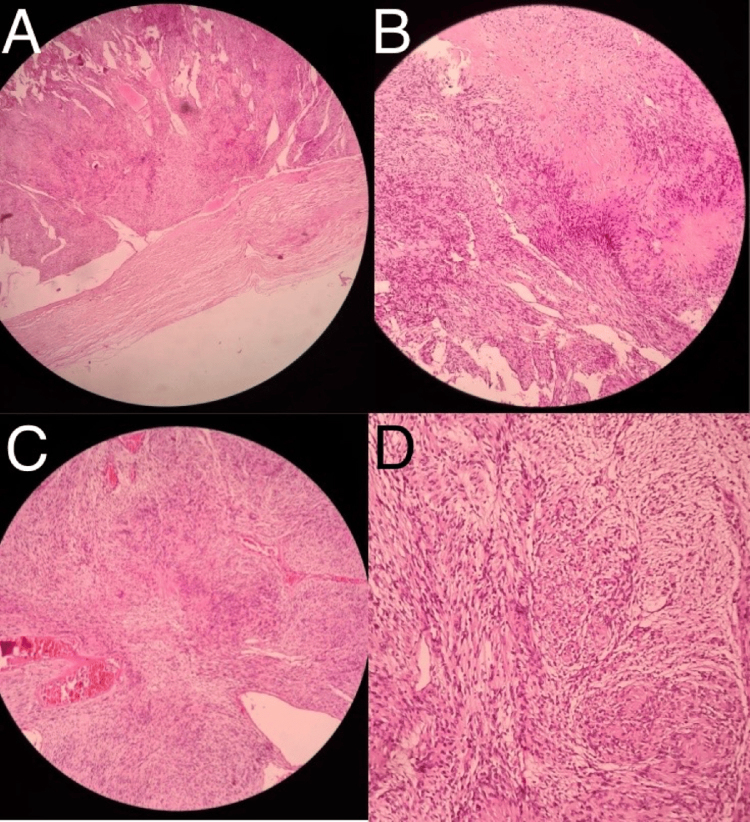
(A) Well encapsulated. (B) Verocay bodies. (C) Mixed Antoni A and B areas (low power). (D) Mixed Antoni A and B areas (high power).

Histopathological evaluation (Fig. 2) of the right thigh mass biopsy revealed a well-encapsulated schwannoma with characteristic Antoni A and B regions, along with Verocay bodies. It was also S-100 positive on immunohistochemistry. Genetic testing was not done as the patient was unable to afford it.

The patient underwent staged surgical interventions due to the multiple schwannomas. The spinal schwannoma was prioritized for surgical removal due to its mass effect and progression of cauda equina syndrome symptoms. A posterior laminectomy from L2 to L4 was performed, and the tumor was dissected from the cauda equina nerve roots. The lesion was partially resected to preserve nerve function, achieving approximately 80% tumor debulking. Intraoperatively, the tumor was firm, encapsulated, and adhered to the nerve roots without frank infiltration.

For symptomatic peripheral schwannomas, the left lower abdominal wall and medial right arm lesions were excised completely. These surgeries were performed 1 month apart from the spinal surgery, as they were not causing significant neurological impairment but contributed to discomfort and pain.

Postoperatively, the motor strength improved to 4/5 on the MRC scale in both lower limbs, and bladder function showed partial recovery. Sensory deficits improved, but hypoesthesia persisted in the right L5-S1 dermatomes. Mild residual weakness remained in the right lower limb, and the patient continued to experience intermittent neuropathic pain, which was managed with pregabalin and NSAIDs.

Histopathological evaluation of the resected spinal mass confirmed it to be schwannoma, showing alternating Antoni A and Antoni B regions, along with Verocay bodies. The tumor cells exhibited elongated nuclei arranged in palisades. Immunohistochemical staining demonstrated strong S-100 positivity, confirming the Schwann cell origin of the neoplasm. The Ki-67 proliferation index was low (<2%), indicating slow-growing behavior. No evidence of malignancy was observed.

At the 6-month follow-up, the patient reported significant improvement in back pain and partial recovery of lower limb strength following spinal decompression surgery. However, mild residual weakness persisted in the right lower limb, and she continued to experience intermittent neuropathic pain, which was managed with pregabalin and NSAIDs. The patient was reassured and advised to return for earlier review if she developed worsening pain, weakness, or new neurological deficits.

## Discussion

Schwannomatosis or neurilemmomatosis are multiple schwannomas without other stigmata of neurofibromatosis NF-1 and NF-2. While Cauda equina schwannomas (CESs) are uncommon benign tumors that usually lead to cauda equina syndrome^[5,6]^.

Evans *et al*^[7]^ reported prevalence of 1 in 126 315 of schwannomatosis. When a patient presents with lower back pain and paresthesias, there are numerous causes of it like lumbosacral muscle strains and sprains, lumbar spondylosis, disk herniations, tumors, spinal stenosis, etc^[8]^. Lower back pain is a common disorder affecting 80% of the world’s population^[9]^.

In particular, diagnosis of schwannomatosis can be very difficult due to its overlapping features with neurofibromatosis^[10]^. Plotkin *et al*^[11]^ updated the diagnostic criteria for schwannomatosis incorporating clinical features, histopathology, and molecular testing focusing on using molecular data. Various molecular bases have been reported at different times like Honda suggested that mutations in NF-2 were responsible for schwannomatosis while MacCollin considered schwannomatosis a separate entity for neurofibromatosis^[12,13]^.

Now the molecular mechanism of schwannomatosis is thought to consist of mutations in two genes *SMARCB1* and *LZTR1*^[14]^. These tumor suppressor genes found on chromosome 22, near the NF2 region, include *SMARCB1* and *LZTR1*. Germline mutations in *SMARCB1* are responsible for 48% of familial and 10% of sporadic schwannomatosis cases, while mutations in *LZTR1* account for 38% of familial and 30% of sporadic schwannomatosis cases^[15]^.

In histopathology, sharp differences are seen between neurofibromatosis and schwannomatosis. Neurofibromas is an encapsulated tumor with an oval to spindle-shaped matrix consisting of all parts associated with nerves like perineurium, fibroblast Schwann cells, and axons^[2,16]^.

While on histopathology schwannomatosis is clearly defined, encased in a capsule, and contains regions with bundles of Schwann cells displaying a spindle-shaped morphology (Antoni A pattern). These areas may either blend into or abruptly transition into other regions that are more loosely structured and microcystic (Antoni B pattern)^[15,17]^.

Usually, the first line of treatment in case of schwannomatosis is surgical resection^[18]^.

In previous literature, very few reports exist on schwannomatosis causing cauda equina syndrome and all being treated by surgical resection. Chen reported a case of a 37-year-old female presented with schwannomatosis causing cauda equina syndrome and treated with tumor resection and showed favorable recovery^[19]^.

Neto *et al*, Saiful Azli *et al*, Domínguez *et al*, and Wierzbicki *et al* reported schwannomatosis precipitating cauda equina syndrome all being treated by surgical resection^[20–23]^. However, Makashova *et al*^[24]^ reported use of targeted therapy with bevacizumab is effective in patients with bilateral schwannomas with a significant risk of hearing loss, along with challenges in controlling intramedullary tumor growth. Prognosis of schwannomatosis is generally well exemplified by one study existing with follow-up of 15 years^[25]^.

The limitation of our study is only 6 months follow-up till now. So the future complications in our case are not yet known.

## Conclusion

Our case emphasizes the necessity of considering schwannomatosis in individuals who present with multiple schwannomas, even when typical NF2 characteristics are not present. The emergence of CES in this patient illustrates the potential for significant neurological complications associated with schwannomatosis, highlighting the importance of prompt surgical intervention. Although surgical removal continues to be the primary treatment for symptomatic tumors, ongoing exploration of targeted therapies and genetic treatments may provide new optimism for those dealing with this rare and complex disorder.

## Data Availability

All the relevant data have been included in the manuscript itself.
